# Microbial Community Dynamics of Soybean (*Glycine max*) Is Affected by Cropping Sequence

**DOI:** 10.3389/fmicb.2021.632280

**Published:** 2021-02-11

**Authors:** Ayooluwa J. Bolaji, Joey C. Wan, Christopher L. Manchur, Yvonne Lawley, Teresa R. de Kievit, W. G. Dilantha Fernando, Mark F. Belmonte

**Affiliations:** ^1^Department of Biological Sciences, University of Manitoba, Winnipeg, MB, Canada; ^2^Department of Plant Science, University of Manitoba, Winnipeg, MB, Canada; ^3^Department of Microbiology, University of Manitoba, Winnipeg, MB, Canada

**Keywords:** canola, corn, crop sequence, phytomicrobiome, soybean

## Abstract

The microbial composition of the rhizosphere soil could be an important determinant of crop yield, pathogen resistance, and other beneficial attributes in plants. However, little is known about the impact of cropping sequences on microbial community dynamics, especially in economically important species like soybean. Using 2-year crop sequences of corn-soybean, canola-soybean, and soybean-soybean, we investigated how crops from the previous growing season influenced the structure of the microbiome in both the bulk soil and soybean rhizosphere. A combination of marker-based Illumina sequencing and bioinformatics analyses was used to show that bacterial species richness and evenness in the soybean rhizosphere soil were similar following canola and soybean compared to a previous corn sequence. However, fungal species richness and evenness remained unaffected by crop sequence. In addition, bacterial and fungal species diversity in both the bulk and soybean rhizosphere soil were not influenced by crop sequence. Lastly, the corn-soybean sequence significantly differed in the relative abundance of certain bacterial and fungal classes in both the soybean rhizosphere and bulk soil. While canola-soybean and a continuous soybean sequence did not, suggesting that a preceding corn sequence may reduce the occurrence of overall bacterial and fungal community members. For the present study, crop sequence impacts bacterial diversity and richness in both the bulk soil and soybean rhizosphere soil whereas fungal diversity and richness are resilient to crop sequence practices. Together, these findings could help drive decision making for annual crop and soil management practices.

## Introduction

Crop rotation is an agricultural management practice used by growers to disrupt the life cycle of pests, increase soil fertility, and suppress the growth of weeds (Cardina et al., 2002; Smith et al., 2008; Rusch et al., 2013; Mcdaniel et al., 2014). Reports show that cropping sequences within the rotation may alter soil microbial communities and confer beneficial effects for improved agronomic performance (Mcdaniel et al., 2014; Samuel Venter et al., 2016). These soil microbial communities enhance plant growth through the secretion of metabolites, mobilization of nutrients, and alleviation of biotic and abiotic stressors (de Garcia et al., 2001; Rijavec and Lapanje, 2016; Tahir et al., 2017). In addition, plants can influence the composition and function of their microbiome by selectively recruiting beneficial microbes from their adjacent environment to the rhizosphere (Berendsen et al., 2012; Thijs et al., 2016). The plant roots of soybean (Sugiyama et al., 2014), Arabidopsis (Chaparro et al., 2014), grape (Novello et al., 2017), and corn (Walters et al., 2018) have all been shown to influence microbial community dynamics. Compared to the bulk soil where plant roots do not penetrate, plants may alter the microbial composition of the rhizosphere. Plant roots may secrete exudates while soil microbes may enhance plant growth, in part, through the mobilization of essential nutrients, manipulating hormone signaling of the plant, and repelling or outcompeting pathogenic microbes (Chaparro et al., 2012; Jacoby et al., 2017). Together, these studies suggest that the microbial composition of the bulk and rhizosphere soil are driven by active selection by the plant root and provide opportunities for exploration.

It has been suggested that the rhizosphere and bulk soil possess different microbial composition as the rhizosphere harbors elevated numbers of active microorganisms than the bulk soil due to increased nutrient availability in the rhizosphere (Benitez et al., 2017; Maarastawi et al., 2018; Liu H. et al., 2019; Liu J. et al., 2019). For example, using maize roots, it was recently reported that the microbiome of the rhizosphere in both conventional and organic systems were more similar than communities from their respective bulk soils (Schmidt et al., 2019). Also, denaturing gradient gel electrophoresis analysis of bulk soil and strawberry rhizosphere samples revealed similar detected bacterial species but with significant differences in their relative proportions. Furthermore, differences in the relative bacterial abundance in rhizosphere samples, when compared to bulk soil, were more pronounced in the second year of the study (Smalla et al., 2001). In soybean, changes in rhizosphere bacterial communities were observed while in contrast, bulk soil bacterial communities showed no significant changes across plant development (Sugiyama et al., 2014). Together, these studies suggest that the initial microbial composition of the rhizosphere is derived from the bulk soil. Alternatively, active selection over time by plant roots results in defined differences in the relative abundance of detected microbes in the rhizosphere compared to the bulk soil.

Recently, a growing number of studies have focused on the effect of cropping sequence on soil microbial community structure using different approaches (Brookes and Barfoot, 2014; Samuel Venter et al., 2016; Schmidt et al., 2019; Agomoh et al., 2020). Cropping sequences involving corn and soybean are well studied and has and been reported to enhance soil stability and fertility compared to continuous cropping of corn or soybean due to the increase of beneficial microbial taxa (Peralta et al., 2018; Song et al., 2018; Agomoh et al., 2020). However, the long-term effects of continuous cropping of both soybean and corn lead to a decrease in microbial species diversity (Peralta et al., 2018; Liu H. et al., 2019; Liu J. et al., 2019; Liu Z. et al., 2019), thus resulting in lower crop yields (Seifert et al., 2017).

While our understanding of a corn-soybean crop sequence on soil microbial dynamics is becoming clearer (Benitez et al., 2017; Chamberlain et al., 2020), little is known about the effects of canola-soybean or continuous soybean sequences on microbial community composition in the bulk and soybean rhizosphere soil. Furthermore, the majority of published work that investigates the impact of cropping sequence regimes on the soil microbiome focused solely on rhizospheric bacterial communities at a single time-point, while few studies have investigated how the fungal community structure and bulk soil respond to cropping sequence practices over time (Granzow et al., 2017; Liu H. et al., 2019; Schmidt et al., 2019; McKenna et al., 2020).

In this study, we characterized the effect of three crop sequence treatments: soybean-soybean, corn-soybean, and canola-soybean on the abundance, species diversity, and community composition of bacterial and fungal populations within the bulk and rhizospheric soils of soybean. It was hypothesized that bacterial and fungal species diversity and community composition would differ based on the crop sequence and that the preceding crop would influence the microbial composition of both the bulk and rhizospheric soils even over the relatively short period of one growing season. We report significant differences in the bulk and rhizosphere soil of bacterial species richness and diversity of the corn-soybean treatment while the bulk and rhizosphere soil microbiome of soybean following canola or soybean itself was unaffected. Also, the relative abundance of certain bacterial and fungal were influenced by crop sequence in our study. Taken together, the results of this study provide an understanding of microbial community dynamics for three different crop sequences.

## Materials and Methods

### Field Site and Experimental Design

The research presented in this article was carried out at the Ian N. Morrison Research Farm located near Carman, Manitoba, Canada (49°29′53N 98°01′47W). The soil sampled for this study was an Orthic Black Chernozem according to the Canadian system of soil classification and was managed using conventional tillage. The experimental design was a randomized complete block with three replicates and three crop sequence treatments. Plots measured 8 × 8 m and soil samples were obtained within the middle 2 × 8 m of the plot. Year one of the experiment consisted of a treatment crop of canola, corn, or soybean and in year two soybean was planted in all treatments such that the 2-year crop sequence treatments were canola-soybean (Ca-S), corn-soybean (C-S), and soybean-soybean (S-S).

### Crop Management

In year one of the crop sequence experiment, canola (cv. 74-44BL) was seeded at a target population of 110 plants m^–2^ with a seed drill and 19 cm row spacing. Corn (cv. DKC26-28RIB) was seeded at a target population of 7 plants m^–2^ using a vacuum planter and 76 cm row spacing. Soybean (cv. 24-10RY) was seeded at a target population of 47 plants m^–2^ with a vacuum planter and 38 cm row spacing. Fertilizers were broadcast and incorporated in spring before planting based on soil test recommendations. Canola received 84 kg N ha^–1^, 69 kg P ha^–1^, and 34 kg S ha^–1^. No fertilizer was applied to soybean, rather granular *Bradyrhizobium japonicum* inoculant was applied in-furrow at 8.0 kg ha^–1^ (Cell-Tech^®^ granular, Novozymes Bio Ag, Saskatoon, Canada). Glyphosate (Roundup WeatherMAX^®^, Bayer Crop Science, Calgary, Canada) was used for post-emergence weed control at recommended rates and timing for each crop as all crop varieties used for the experiment were herbicide-tolerant. After the harvest of each crop type in year one, crop residues were incorporated into the soil using tillage in the fall.

In year two of the study, soybean (cv. 24-10RY) was seeded at a target population of 40 plants m^–2^ with a vacuum planter and 76 cm row spacing. *Bradyrhizobium japonicum* inoculant was applied in-furrow at 7.3 kg ha^–1^ (Nodulator^®^ granular, BASF, Mississauga, Canada). Glyphosate (Roundup WeatherMAX^®^, Bayer Crop Science, Calgary, Canada) was used for post-emergence weed control at recommended rates and timing as the soybean variety used for this experiment was herbicide-tolerant.

### Bulk Soil and Soybean Rhizosphere Soil Sample Collection

**FIGURE 1:**
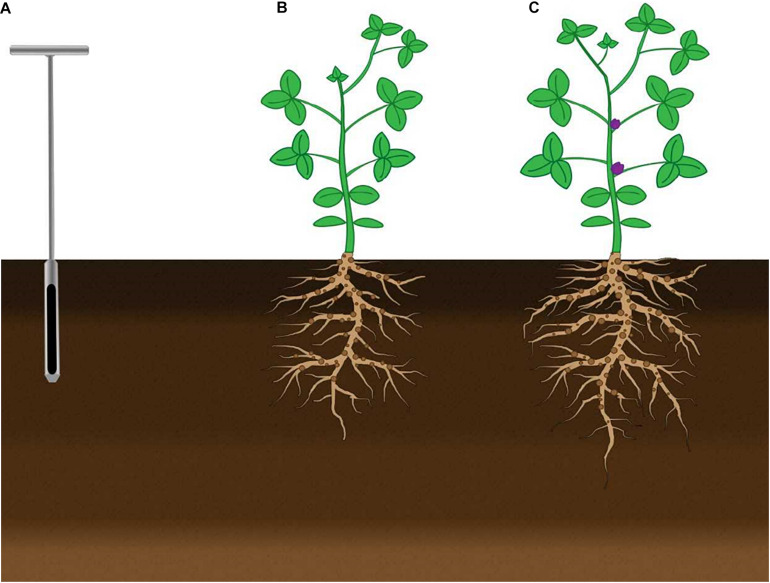
Schematic figure of the timing of sample collection from bulk soil and soybean rhizosphere soil for all crop sequences. Bulk soil and rhizosphere soil samples were collected at a depth of 0–20 cm. **(A)** Soil was collected from bulk soil using a soil probe before seeding soybean. Sampling of bulk soil in the inter-row area between soybean plants and rhizosphere soil of soybean at the **(B)** V6 and **(C)** R1 growth stages.

Soil samples for microbial community analyses were collected at three different stages of crop development: Pre-seeding, V6, and R1 (Figure 1). Bulk soil samples were collected from 0 to 20 cm from areas between plant rows using a soil probe. Five samples were randomly collected per plot and pooled together to make a single biological replicate. This approach was repeated in three different plots for a total of three biological replicates. Soil samples were flash-frozen and stored at −80°C until processing. Rhizosphere soil samples were collected from the soil adhered to the roots of soybean. Five root systems were isolated using a spade from 0 to 20 cm and pooled to generate one biological replicate; with a total of 15 plants and three biological replicates. Root samples were flash-frozen and stored at −80°C until needed; at which point the samples were slowly thawed on ice and rhizosphere soil was brushed off with sterile paintbrushes and collected for analysis.

### DNA Extraction, Library Synthesis, and Illumina Sequencing

Total genomic DNA was extracted from 0.5 g of soil using the Qiagen DNeasy PowerSoil Kit (Qiagen, Hilden, Germany) according to the manufacturer’s protocol. Extracted template DNA was quantified using the NanoDrop 2100 Spectrophotometer and the Quant-iT dsDNA PicoGreen Assay (Invitrogen, Carlsbad, CA, United States), and the NanoDrop 3300 Fluro-spectrophotometer. Both bacterial (V4) and fungal (ITS) libraries were synthesized following the Earth Microbiome Project protocol^1^. Briefly, the V4 hyper-variable region of the bacterial and archaeal 16S rRNA gene was amplified using bar-coded 515F/806R primers (Caporaso et al., 2011, 2012). Targeted metagenomics was performed with Platinum Hot Start PCR II Mix and the following thermocycling conditions: 94°C for 3 min, 35 cycles of 94°C for 45 s, 50°C for 60 s, 72°C for 90 s, and 72°C for 10 min. Sample PCR amplification was carried out in triplicate to ensure sufficient product for downstream experiments. These triplicates were pooled together, and gel electrophoresis was performed to confirm the proper amplification and absence of unintended PCR products. The ITS1 and ITS2 sequences were amplified with barcoded ITS1/ITS2 primers (Bokulich and Mills, 2013). PCR amplification was performed as previously with the following modifications: 94°C for 1 min, 35 cycles of 94°C for 30 s, 52°C for 30 s, 68°C for 30 s, and 6°C for 10 min. Both V4 and ITS libraries were cleaned with the Qiagen UltraClean 96 PCR Cleanup Kit (Qiagen, Hilden, Germany) according to the manufacturer’s recommended protocol. Following cleanup, library concentration was measured with the Quant-iT PicoGreen dsDNA Assay Kit and the NanoDrop 3300 Flurospectrophotometer. Samples were pooled based on concentration and the size distribution of sequencing pools was determined on an Agilent DNA 1000 Chip and Agilent 2100 Bioanalyzer. Library pools, multiplex index of 60, were sent to Genome Quebec and 100 bp paired-end sequencing was conducted on the Illumina HiSeq 4000.

### Sequence Processing

Microbiome sequencing data were analyzed for all crop sequences using the Quantitative Insights Into Microbial Ecology (QIIME) v2.1 suite (Bolyen et al., 2019). First, raw sequencing reads were subsampled with SeqTK^2^ to a depth of 150,000 reads per sample. Briefly, demultiplexed sequences and associated metadata were imported into QIIME via q2-import. Imported sequences were quality filtered, trimming the first five nucleotide reads and reads that fell below the quality threshold, using the q2-demux plugin followed by sequence denoising with DADA2 via q2-dada2 (Callahan et al., 2016). Identified amplicon sequence variants (ASVs) were aligned with mafft (via q2-alignment) and FastTree (via q2-phylogeny) and used to construct phylogenetic trees (Katoh, 2002; Price et al., 2010).

### Data Analysis

Biodiversity metrics were calculated on rarefied samples that were subsampled to 14000 sequences without replacement generating alpha diversity values – observed operational taxonomic units (OTUs), Faith’s Phylogenetic Diversity (FPD-measures of microbiome diversity), Shannon’s Index Diversity and beta diversity values – weighted and unweighted UniFrac, Bray-Curtis Dissimilarity (for bacterial and fungal communities, and principal coordinates analysis via q2-diversity plugin) (Faith, 1992; Lozupone and Knight, 2005; Lozupone et al., 2007). Alpha rarefication, a measure of observed OTUs against sequence count, was performed to ensure that sufficient sequencing depth was achieved to capture the complete biodiversity of the samples (Willis, 2019), which determined that the sampling depth of 14,000 was sufficient to capture the biological diversity present in the samples. Analysis of the composition of the microbiome (ANCOM) was used to identify differentially abundant taxa between bulk soil and rhizosphere soil samples (Supplementary Figure 4; Mandal et al., 2015). Based on the Bray–Curtis dissimilarity between samples, a permutational multivariate analysis of variance (perMANOVA) was conducted using a permutation test with pseudo-F ratio (permutations = 999, sample size = 30 per sample and group = 3 for crop sequences) (Oksanen et al., 2018) to test the community composition change based on crop sequence and the overall effects on crop sequence on the community composition. To understand community composition differences between treatments at each time point, perMANOVA was conducted for each crop sequence. In assessing the bacterial and fungal community composition in the soil, clustering of OTUs was performed at a 99% level of similarity and OTUs with low occurrences (minimum counts of 2500) were discarded for all crop sequences at the pre-seeding, V6, and R1 time-points. Taxonomy was assigned to ASVs via q2-feature-classifier (Bokulich et al., 2018) sklearn naïve Bayes taxonomy classifier against the Greengenes 13_5 99% OTU reference sequences and UNITE 8.2 97% Dynamic reference sequences for bacterial and fungal taxonomic assignment, respectively (DeSantis et al., 2006; Nilsson et al., 2019). Proportional phyla and class bar plots were generated using q2-bar plots; detected taxa were averaged across three biological replicates. The Kruskal-Wallis test was used to measure species diversity for all treatments in bulk and rhizosphere soil and was calculated using QIIME v2.1 suite. Multiple comparison tests of taxa differentially abundant across treatments were performed using the Kruskal-Wallis test. A differential analysis of the OTUs relative abundances was carried out in QIIME v2.1 suite, using the percentile abundances of features by group table generated from the ANCOM analysis using the class level data. Differential abundance was calculated using abundance counts. All tests considered *P* < 0.05 as the significance threshold unless stated otherwise.

## Results

### Effect of Crop Sequence on Bacterial Species Diversity in the Bulk Soil

**FIGURE 2:**
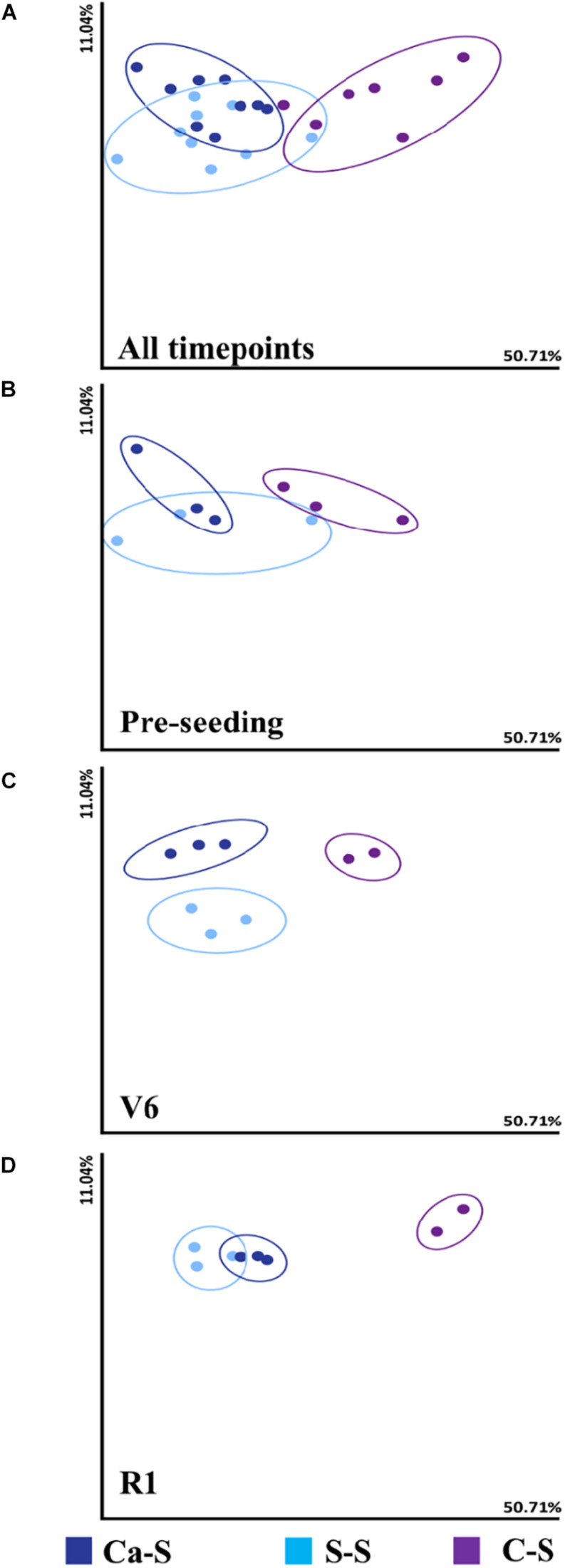
Influence of crop sequence on bacterial species diversity in the bulk soil Principal coordinate analysis (PCoA), represented by the beta diversity metric – Weighted UniFrac, comparing the effect of crop sequence on microbial species diversity among treatments at all time points in the bulk soil. For each axis, the percent of variation is indicated. Dark blue dots, canola-soybean sequence (Ca-S); light blue dots, soybean-soybean sequence (S-S); purple dots, corn-soybean sequence (C-S). Each dot represents the average of three biological replicates and each biological replicate represents five pooled core soil samples. Comparison of cumulative bacterial species diversity for all sequences collect at **(A)** all time-points **(B)** pre-seeding **(C)** V6, and **(D)** R1 soybean growth stages. Because of similarities in the data, some data points are overlapping.

To determine the effect of crop sequence on bacterial species diversity, we first analyzed diversity metrics in the bulk soil at three time points: pre-seeding, V6, and R1 using weighted UniFrac beta diversity metric (Figure 2). Soil sample was obtained from three biological replicates for each time-point. A measure of species diversity was calculated for the different sequences and compared to each other with a pairwise Kruskal-Wallis test. The analysis of bacterial composition did not differ significantly for the S-S sequence nor the Ca-S sequence in the bulk soil. We observed significant differences in the alpha diversity of the C-S sequence (*P* < 0.05) (Figure 2A). At the pre-seeding stage (Figure 2B), V6 (Figure 2C), and R1 (Figure 2D) time-points, the difference observed for the Ca-S and S-S sequences was not significant (Figure 2A). However, the species diversity within the C-S sequence differed significantly less (*P* < 0.05) compared to the other two sequences (Figure 2A). The diversity metrics in the rhizosphere soil were also measured and similar results to the bulk soil were obtained, where the bacterial diversity in the C-S sequence differed significantly from the Ca-S and S-S sequences. Taken together, the bacterial species diversity in the C-S sequence is different from Ca-S and continuous S-S sequence in both the bulk and rhizosphere soil at all time-points.

### Effect of Crop Sequence on Fungal Species Diversity

**FIGURE 3:**
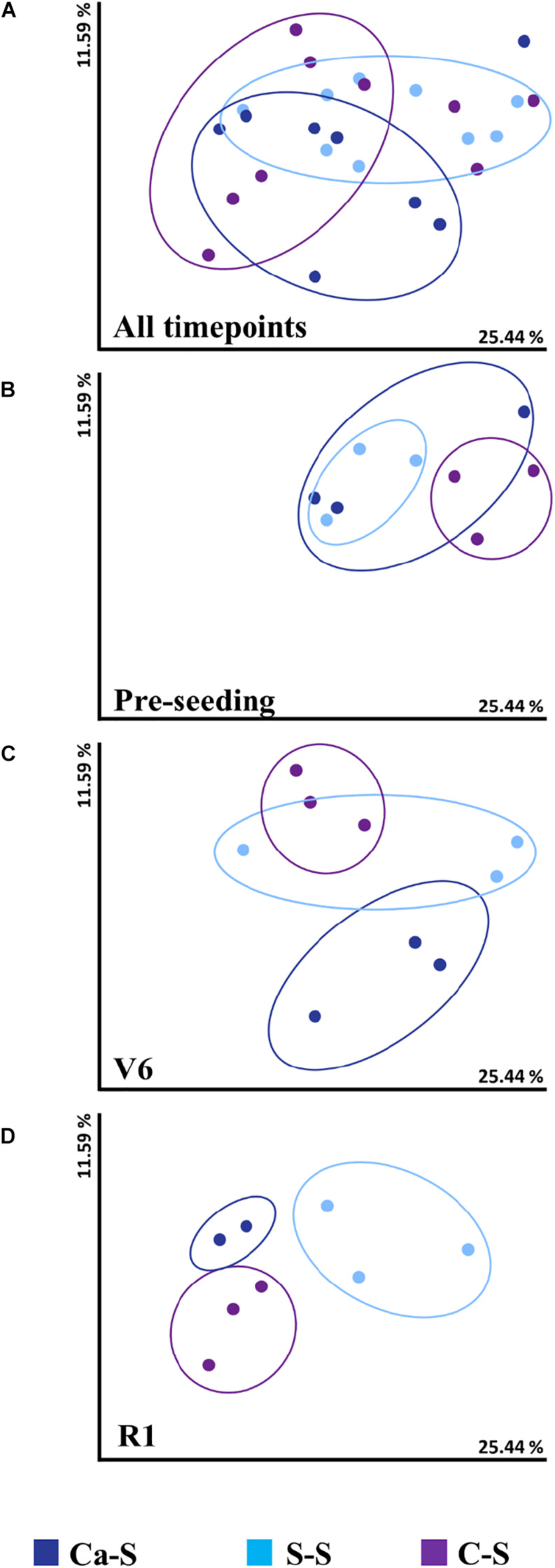
Influence of crop sequence on fungal species diversity in the bulk soil Principal coordinate analysis (PCoA), represented by the beta diversity metric – Weighted UniFrac, comparing the effect of crop sequence on microbial species diversity among treatments at all time points in the bulk soil. For each axis, the percent of variation is indicated. Dark blue dots, canola-soybean sequence (Ca-S); light blue dots, soybean-soybean sequence (S-S); purple dots, corn-soybean sequence (C-S). Each dot represents the average of three biological replicates and each biological replicate represents five pooled core soil samples. Comparison of cumulative fungal species diversity for all sequences collect at **(A)** all time-points **(B)** pre-seeding **(C)** V6, and **(D)** R1 soybean growth stages. Because of similarities in the data, some data points are overlapping.

Given that the bacterial species diversity was influenced by crop sequence in the bulk soil, similar tools were used to assess fungal species diversity for all three crop sequences (Figure 3). Fungal species diversity did not differ significantly (*P* > 0.05) among the sequences at the cumulative, pre-seeding, and V6 time-points (Figures 3A–C). At the R1 time-point, fungal diversity differed significantly (*P* < 0.05) for the S-S sequence compared to the C-S and Ca-S sequence (Figure 3D). However, the differences in fungal species diversity observed for all crop sequences at the R1 time-point did not lead to changes in global fungal diversity when samples were cumulated (Figure 3A).

### Estimating Microbial Community Structure Based on Crop Sequence

**FIGURE 4:**
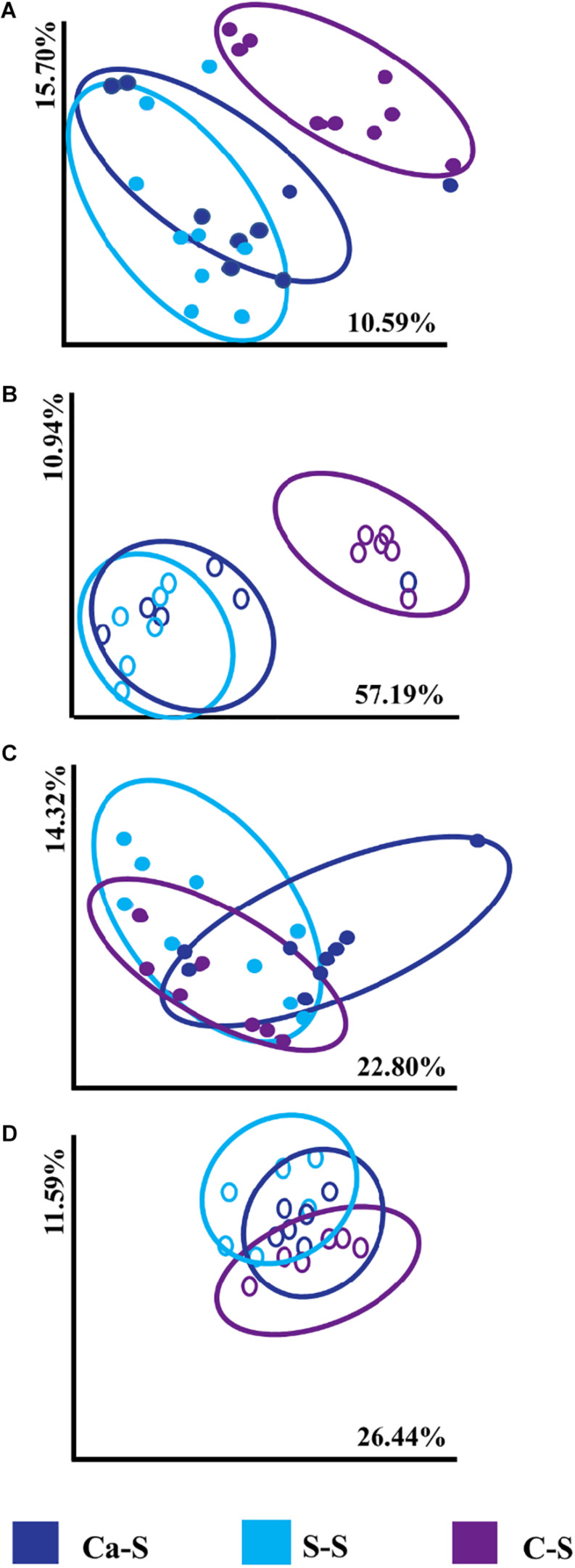
Principal coordinate analysis (PCoA), represented by Bray-Curtis dissimilarities between samples and calculated using the 16S rRNA amplicon sequences for bacterial samples and the ITS1 and ITS2 amplicon sequences of fungal samples. Soils were obtained from the Ca-S, S-S, and C-S crop sequences in the bulk and rhizosphere soil for all time-points. For each axis, the percent of variation is indicated. The dark blue filled and open circles represent the canola-soybean sequence (Ca-S), light blue filled and open circles are the soybean-soybean sequence (S-S) and the purple filled and open circles are the corn-soybean sequence (C-S). Each dot represents the average of three biological replicates and each biological replicate represents five pooled core soil samples. Comparison of cumulative Bray-Curtis dissimilarities for panel **(A)** bacterial in the bulk soil **(B)** bacterial in the rhizosphere soil **(C)** fungal in the bulk soil **(D)** fungal in the rhizosphere soil.

We next studied microbial community structure in response to crop sequence using the Bray-Curtis dissimilarity metric in response to different crop sequences in the bulk soil and the soybean rhizosphere for both bacteria and fungal communities (Figure 4). Bray-Curtis dissimilarity was used since this tool provides a quantitative measure of the abundance of organisms present in the studied populations. Our data show that bacterial community structure in the annual Ca-S sequence and the continuously cropped S-S sequence tended to cluster together compared to the annual C-S sequence which was significantly less from S-S [PERMANOVA *F* = 6.006, *P* = 0.001] and Ca-S [PERMANOVA F = 3.027, *P* = 0.001] sequences for both bulk and rhizosphere soils (Figures 4A,B). Fungal community structure and composition showed all treatments clustered together regardless of crop sequence (Figures 4C,D). Under our field conditions, PERMANOVA revealed significant differences of bacterial community structure in the C-S sequence compared to the Ca-S and S-S sequences in the bulk and rhizosphere soil (Supplementary Table 1) while the fungal community structure was not affected by crop sequence (Supplementary Table 2).

### The Effect of Crop Sequence on Bulk Soil and Soybean Rhizosphere Microbial Species Diversity

**FIGURE 5:**
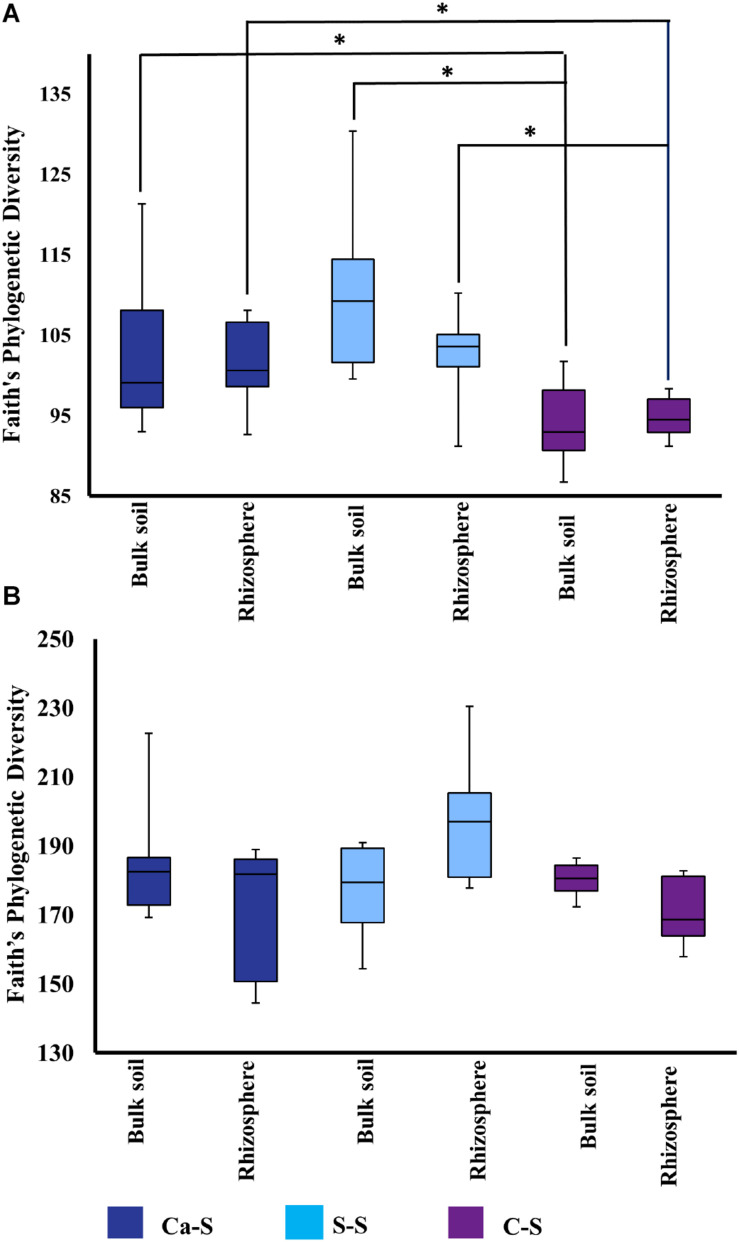
Quantitative comparisons of bacterial species diversity represented by the alpha diversity metric – Faith’s Phylogenetic Diversity –in the bulk soil and rhizosphere soil at all time-points. The median as well as the 25th and 75th percentile of the samples is presented, individual data points outside of this range are given as mean ± standard deviation of three replicate samples. Significant differences due to crop sequences within one compartment (bulk soil or rhizosphere) are noted. The dark blue boxplot represents the canola-soybean sequence (Ca-S), light blue boxplots are the soybean-soybean sequence (S-S) and the purple boxplots are the corn-soybean sequence (C-S). **(A)** Comparison of cumulative phylogenetic diversity of the bacterial community in the bulk soil and rhizosphere soil treatments. **(B)** Comparison of cumulative phylogenetic diversity of the fungal community in bulk soil and rhizosphere soil for all crop sequences and all time points. Statistical significance was analyzed by the Kruskal-Wallis test. Asterisks indicate statistically significance (*P* < 0.05).

Having shown that bacterial species richness of the bulk soil was influenced by crop sequence treatment, while fungal species richness was not, we next studied possible differences in microbial diversity in the rhizosphere soil compared to the bulk soil. Using Faith’s Phylogenetic Diversity (FPD), numerical quantifications were then studied for species richness (Figure 5 and Supplementary Figure 1). Microbial species richness metrics for the bacteria samples were significantly different (*P* < 0.05) in the C-S crop sequence compared to the Ca-S and S-S crop sequences for both the bulk and rhizosphere soils (Figure 5A and Supplementary Figure 1A) while, the fungal samples were not significantly different across any crop sequence (Figure 5B and Supplementary Figure 1B). Within all the crop sequences, bulk soil and rhizosphere soil samples did not show any significant differences (*P* > 0.05) (Figures 5A,B).

### Global Soil Microbial Richness in Response to Crop Sequence

**FIGURE 6:**
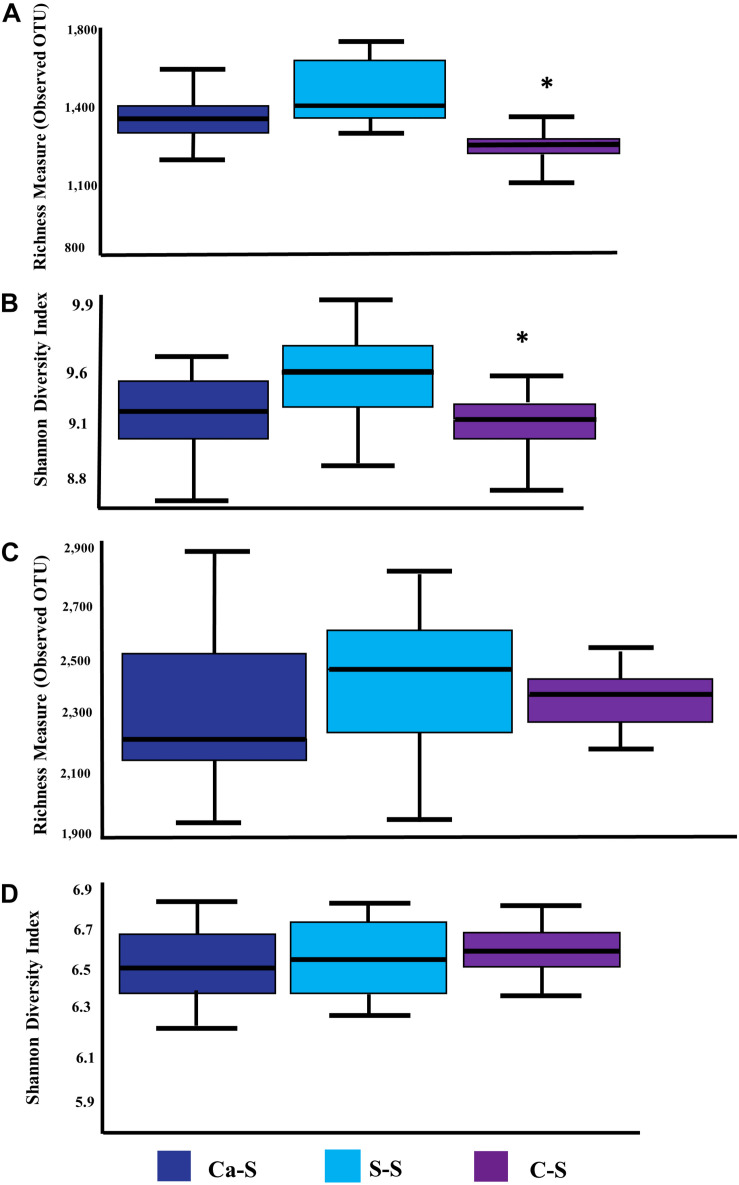
Quantitative comparisons of global bacterial and fungal species richness. The median as well as the 25th and 75th percentile of the samples is presented, individual data points outside of this range are given as mean ± standard deviation of three replicate samples. Significant differences due to crop sequences are noted with an asterisk. The dark blue boxplot represents the canola-soybean sequence (Ca-S), light blue boxplots are the soybean-soybean sequence (S-S) and the purple boxplots are the corn-soybean sequence (C-S). Quantitative comparison of cumulative bulk soil and rhizosphere samples of: **(A)** bacterial species richness measure of observed OTUs **(B)** bacterial Shannon Index Diversity measure of alpha diversity **(C)** fungal species richness measure of observed OTUs **(D)** fungal Shannon Index Diversity measure of alpha diversity. Statistical significance was analyzed by the Kruskal-Wallis test. Asterisks indicate statistically significance (*P* < 0.05).

Having shown that bacterial species diversity of the bulk and rhizosphere soils were influenced by crop sequence treatment, while fungal species diversity was not, we next assessed possible differences in microbial richness in the soybean rhizosphere compared to the bulk soil. Figure 6 shows the number of observed species and Shannon’s Index Diversity as a measure of soil microbial richness. Global microbial species richness metrics for the bacteria samples were significantly different (*P* < 0.05) in the C-S crop sequence compared to the Ca-S and S-S crop sequences (Figures 6A,B) while, the fungal samples were not significantly different for all crop sequences (Figures 6C,D).

### The Effect of Crop Sequence on Bacterial Community Composition in the Bulk Soil and Rhizosphere Soil

**FIGURE 7:**
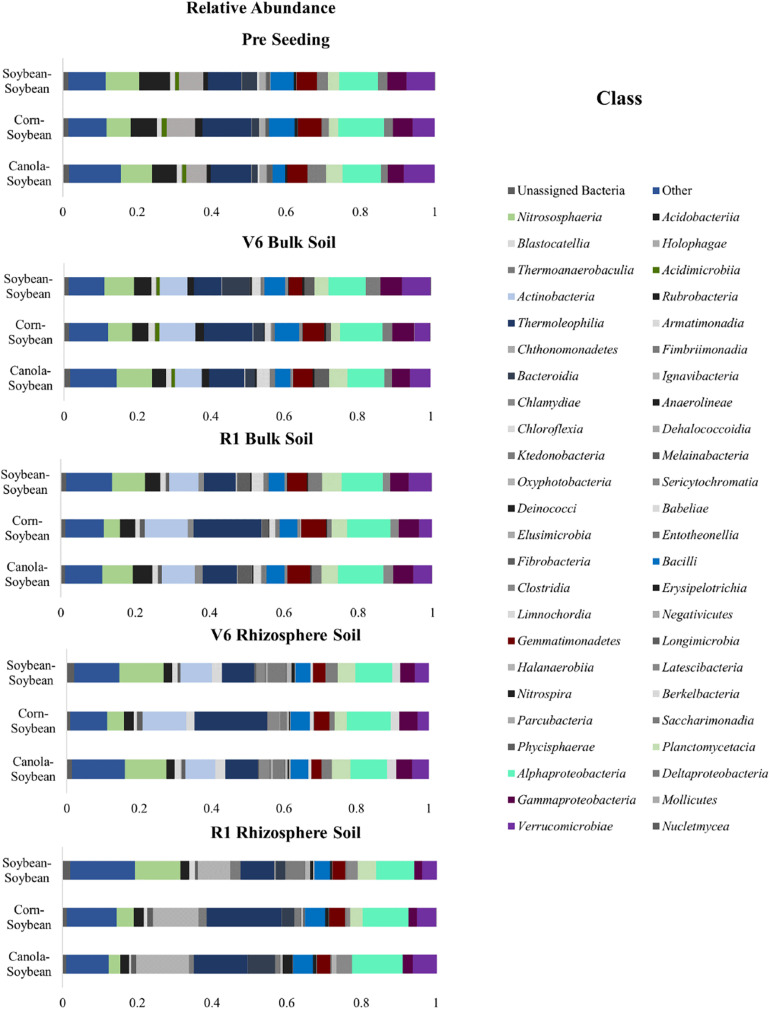
Stacked bar plots of bacterial taxonomic composition showing shifts in bacterial classes in response to crop sequence treatments, during the soybean growing season in the bulk soil and soybean rhizosphere soil. Each bar represents an average of three biological replicates. OTUs with similar species-level identity at 99% similarity in the Greengenes database are merged here. Taxa with less than 1% mean relative abundance across the samples studied are combined and shown as Others. Bar plots of bulk soil treatments of the relative abundance at the pre-seeding, V6, and R1 growth stages; as well as bar plots of the soybean rhizosphere soil of the relative abundance at the V6 and R1 growth stages are presented.

The bacterial community structure of the bulk soil was studied for all sequences at the pre-seeding, V6, and R1 growth stages (Figure 7 and Supplementary Figure 2). We then investigated the relative abundance of the mean values to determine the bacterial community composition; for the pre-seeding stage, at the phyla and class levels. At the phyla level, the A*ctinobacteria* (18–25%), *Proteobacteria* (17–20%), and *Acidobacteria* (15–17%) were the predominant taxa in all three crop sequences (Supplementary Figure 2A). While bacteria abundance of the bacterial community in the bulk soil at the V6 time-point revealed that the bacteria of the *Actinobacteria* comprised 19–28%, *Proteobacteria* comprised 17–20% and *Acidobacteria* comprised 13–15% (Supplementary Figure 2A). At the R1 time-point, the *Actinobacteria* (20–33%), *Proteobacteria* (18–20%), and *Acidobacteria* (11–15%) remained the predominant bacteria phyla (Figure 7). However, a significant increase in the *Actinobacteria* population (*P* < 0.05) was observed in the R1 time-point for the corn-soybean sequence (from 28% in V6 to 33% in R1). The relative abundance of A*ctinobacteria, Proteobacteria*, and *Acidobacteria* in the bulk soil was similar in the Ca-S and S-S sequences for all time-points (*P* > 0.05) but differed significantly to the C-S sequence (*P* < 0.05).

Next, we studied the taxonomy of the bulk soil and soybean rhizosphere sequence data at the class level across all time points (Figure 7). Of the forty-eight assigned bacterial classes, *Gammaproteobacteria*, *Verrucomicrobiae, Acidobacteria*, *Thermoleophilia*, and *Alphaproteobacteria* were most dominant. At the pre-seeding time-point, bacteria belonging to the class *Gammaproteobacteria* (4–5%), *Verrucomicrobiae* (6–8%), *Acidobacteria* (7–8%) *Thermoleophilia* (9–13%), and *Alphaproteobacteria* (10–12%) were the most abundant bacterial populations (Figure 7). At the V6 growth stage, *Gammaproteobacteria* (7–10%), *Verrucomicrobiae* (4–6%), *Acidobacteria* (4–5%), *Thermoleophilia* (8–13%) and *Alphaproteobacteria* (10–11%) were the most abundant bacteria (Figure 7). While at the R1 growth stage, *Verrucomicrobiae* (4–6%), *Gammaproteobacteria* (5–7%), *Actinobacteria* (8–12%), *Thermoleophilia* (9–18%), and *Alphaproteobacteria* (11–12%) were some of the most abundant bacterial populations. At the class level, a significant increase in the *Thermoleophilia* was observed for the C-S sequence compared to the Ca-S and S-S sequences (Figure 7). Taken together, at the class level and in the bulk soil, a significant increase (*P* < 0.05) in relative abundance of *Thermoleophilia*, and *Actinobacteria* was observed for the C-S sequence compared to the Ca-S and S-S sequences (Figure 7). ANCOM analysis of differentially abundant taxa at the class level (Supplementary Figure 4A and Supplementary Tables 4, 5) revealed *Actinobacteria* were differentially abundant in the C-S sequence.

The community composition in the soybean rhizosphere was also studied at the V6 and R1 time-points (Supplementary Figure 2B). At the V6 time-point, the predominant bacterial phyla had the same relative abundance for all sequences with the A*ctinobacteria* comprising 22%, *Proteobacteria* comprising 17%, and *Acidobacteria* comprising 14% of the bacteria community composition (Supplementary Figure 2). At the R1 time-point, in the rhizosphere soil, the *Actinobacteria* population increased to 32% for the canola-soybean sequence and 37% for the corn-soybean sequence (Supplementary Figure 2). An increase in the population of the *Proteobacteria* phyla was also observed in the R1 time-point for the canola-soybean and the corn-soybean sequences. Conversely, the *Acidobacteria* population decreased in all crop sequence treatments at the R1 time-point (Supplementary Figure 2).

We then studied the relative abundance of bacteria in the soybean rhizosphere soil at the class level. At the V6 time-point, the dominant bacteria belonged to the class *Bacilli* (4–5%), *Actinobacteria* (9–12%), *Thermoleophilia* (9–20%), and *Alphaproteobacteria* (10–12%) for all crop sequences (Figure 7). The relative abundance of *Thermoleophilia* in the C-S sequence differed significantly from the Ca-S and S-S sequences (*P* < 0.05). At the R1 time-point, the dominant taxa were *Bacilli* (4–5%), *Actinobacteria* (9–14%), *Thermoleophilia* (9–20%), *Alphaproteobacteria* (4–14%), and *Deltaproteobacteria* (3–12%). Both the *Thermoleophila* (*Actinobacteria*) and *Alphaproteobacteria* (*Proteobacteria*) differed significantly at the V6 time-point in the soybean rhizosphere soil for the C-S sequence compared to the Ca-S and S-S sequences. Taken together, in the rhizosphere soil, the differences observed in the global bacteria phyla community composition of the C-S sequence compared to the Ca-S and S-S were different at both the V6 and R1 time-points at the class level (Figure 7). In the rhizosphere, the relative abundance of *Thermoleophila* differed in the C-S sequence compared to the Ca-S and S-S sequences (Figure 7). While *Alphaproteobacteria* decreased significantly at the R1 time-point in the C-S sequence compared to both the Ca-S and S-S sequences (Figure 7).

### The Effect of Crop Sequence on Fungal Community Composition in the Bulk Soil and Soybean Rhizosphere

**FIGURE 8:**
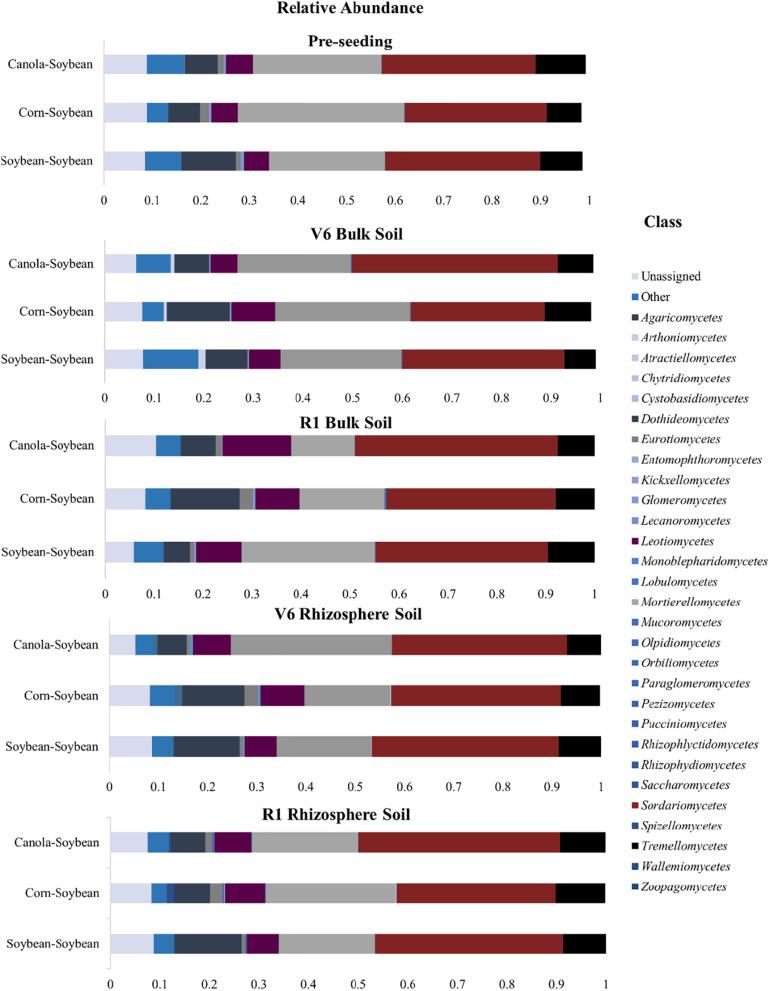
Stacked bar plots of fungal taxonomic composition showing shifts in fungal class in response to crop sequence and over the soybean growing season in the bulk soil and rhizosphere soil. Each bar represents an average of three biological replicates. OTUs with similar species-level identity at 97% similarity in the UNITE database are merged. Taxa with less than 1% mean relative abundance across the samples studied are combined and shown as Others. Bar plots of bulk soil treatments of the relative abundance at the pre-seeding, V6, and R1 growth stages; as well as bar plots of the soybean rhizosphere soil of the relative abundance at the V6 and R1 growth stages are presented.

Fungi belonging to the phyla *Ascomycota, Mortierellomycota*, and *Basidiomycota* were the predominant taxa for all crop sequence treatments and at all time-points (Figure 8). In the bulk soil, at the pre-seeding stage, the *Ascomycota* (51–53%), *Mortierellomycota* (28–33%), and *Basidiomycota* (9–12%) comprised most of the fungal community composition (Supplementary Figure 3). At the V6 time-point, an increase in the *Ascomycota* phyla was observed for all crop sequence treatments, with this phyla comprising 56–63% of the relative abundance of fungi population, this is reflected in the class level with an increase in the population of *Sordariomycetes* at the V6 stage (Figure 8), while *Mortierellomycota* comprised 24–28% of the relative abundance of fungi population and *Basidiomycota* comprised 8–9% of the relative abundance of fungi population (Supplementary Figure 3A). At the R1 time-point, the *Ascomycota* made up 54–63% of the fungal population with *Mortierellomycota* and *Basidiomycota* phyla comprising 15–28 and 9–13%, respectively of the relative abundance of fungi population (Supplementary Figure 3A). A decrease in the abundance of the *Mortierellomycota* was observed for the annual Ca-S and C-S sequences at the pre-seeding and the R1 time-points (Supplementary Figure 3A). All phyla differences observed in the bulk soil in comparison to the soybean rhizosphere were not significant (*P* > 0.05).

At the class level, fungi belonging to *Mortierellomycetes*, *Sordariomycetes*, and *Tremellomycetes* were the dominant fungi for all time-points in the bulk soil out of the 25 assigned fungi classes (excluding the unassigned class) (Figure 8). At the pre-seeding timepoint, the *Mortierellomycetes* (24–36%), *Sordariomycetes* (32–30%), and *Tremellomycetes* (7–10%) were some of the most abundant fungal populations (Figure 8). At the pre-seeding stage, the C-S sequence had significantly more *Mortierellomycetes* than the Ca-S and S-S sequences (*P* < 0.05). At the V6 stage, *Mortierellomycetes* (23–27%), *Sordariomycetes* (23–27%), and *Tremellomycetes* (7–10%) made up some of the most abundant fungi in our dataset (Figure 8). For the V6 timepoint, no significant differences were observed for any of the fungi classes for the different crop sequences. At the R1 time-point, *Mortierellomycetes* (13–27%), *Sordariomycetes* (36–41%), and *Tremellomycetes* (8–10%) comprised some of the most abundant fungi in our samples. The composition of *Mortierellomycetes* was significantly more abundant in the S-S sequence compared to both C-S and Ca-S crop sequences (Figure 8).

In the soybean rhizosphere soil, fungi belonging to the phyla *Ascomycota, Mortierellomycota*, and *Basidiomycota* were the predominant taxa for all three crop sequence treatments (Supplementary Figure 4). At the V6 time-point, the *Ascomycota* dominated 49–53% of the relative abundance of fungi population, *Mortierellomycota* comprised 23–36% of the relative abundance of fungi population, and *Basidiomycota* comprised 20–28% of the relative abundance of fungi population. The C-S sequence had more *Basidiomycota* fungi than the Ca-S and S-S sequences at the V6 growth stage (Supplementary Figure 4). At the R1 growth stage, we observed an increase in the *Ascomycota* population from 57 to 63% while the *Mortierellomycota* decreased from 20 to 23%, and the *Basidiomycota* remained at 10% of the fungal population in the rhizosphere soil (Supplementary Figure 4). In the C-S sequence, at the R1 time-point, the population of the phyla, *Basidiomycota* decreased by 50% compared to the Ca-S and S-S crop sequences (Figure 8). However, in the rhizosphere soil samples, all differences observed in the global fungal community composition were not significant (*P* > 0.05).

At the class level, the dominant fungi in the soybean rhizosphere were *Mortierellomycetes*, *Sordariomycetes*, *Tremellomycetes*, and *Leotiomycetes* (Supplementary Figure 4D and Supplementary Table 6). At the pre-seeding time-point, *Mortierellomycetes* (17–33%), *Sordariomycetes* (35–38%), *Leotiomycetes* (7–10%), and *Tremellomycetes* (7–8%) were found to abundant in our samples. The Ca-S sequence had significantly more *Mortierellomycetes* (*P* < 0.05) compared to the C-S and S-S sequences (Supplementary Figure 3B). At the R1 time-point, *Mortierellomycetes* (21–26%), *Sordariomycetes* (32–41%), *Leotiomycetes* comprised (6–8%), and *Tremellomycetes* (9%) were some of the most abundant fungal populations in our study. The C-S sequence had significantly fewer *Sordariomycetes* compared to the Ca-S and S-S sequences (Figure 8). Taken together, at the phyla level, crop sequence did not significantly influence the fungal community composition in either the bulk soil or the soybean rhizosphere (Supplementary Figure 3B). However, at the class level, the composition of *Mortierellomycetes* was significantly more abundant in the S-S sequence compared to both C-S and Ca-S crop sequences (Figure 8). In the rhizosphere soil, the C-S sequence had significantly fewer *Sordariomycetes* compared to the Ca-S and S-S sequences (Figure 8 and Supplementary Table 6). Thus, differences in the relative abundance of fungal community structure was observed when fungal class levels were studied.

## Discussion

Cropping sequences within a crop rotation is a management practice that enhances diversity, breaks pest cycles, cycles nutrients, and enhances crop productivity (Cardina et al., 2002; Peralta et al., 2018). We investigated how endogenous microbial communities respond over a 2-year crop sequence study that included: corn-soybean (C-S), canola-soybean (Ca-S), and soybean-soybean (S-S) sequences. We investigated how crops from the previous growing season influenced the structure of the microbiome in both the bulk and rhizosphere soil during the soybean growing season. Our results show that bacterial community diversity and richness during the soybean growing season was influenced by a preceding corn crop sequence while the fungal community diversity and richness remained unchanged for all crop sequences. The relative abundance of certain bacteria and fungal taxa were affected by the preceding crop sequence. However, we report that crop sequence did not influence microbial community diversity in the bulk and soybean rhizosphere soil of our study system.

### Crop Sequence Influences Bacterial Community Diversity and Richness in the Bulk Soil and Soybean Rhizosphere

The effect of crop sequence on both bacterial diversity and richness were examined for all three crop sequences during the soybean growing season. The Ca-S and S-S treatments had similar bacterial species diversity and richness but they both differed compared to the C-S crop sequence. Differences in bacterial diversity and community composition observed between the crop sequences agree with previous studies that report distinct differences in the composition of soil bacteria communities between continuously cropped soybean and crop rotation treatments of corn-soybean (Ashworth et al., 2017; Chamberlain et al., 2020). These previous studies in addition to our own suggest that bacterial diversity and richness are influenced by the preceding crop in agricultural soils (Ashworth et al., 2017; Chamberlain et al., 2020). To our knowledge, the present study is the first to investigate the microbial community composition of an annual canola-soybean sequence compared to a continuous soybean-soybean sequence. However, the finding that a Ca-S crop sequence had a similar effect on bacteria community diversity as the S-S sequence agrees with previous studies that show bacterial diversity and richness is non-host-specific (Lay et al., 2018). For example, when canola was grown after field peas and wheat in a cropping sequence, data showed that bacteria in the canola and pea rhizosphere were similar, yet different from the bacteria community found in the wheat rhizosphere; although no explanation was offered (Lay et al., 2018). However, experiments that investigate why a canola-soybean and soybean-soybean sequences have similar effects on bacterial diversity and richness are required to determine the biological interactions present between soybean and canola bacterial communities.

The differences in bacterial species diversity and richness observed in the C-S sequence persisted throughout the growing season. In the current study, we conclude that growing soybean in two sequential years after canola was not capable of restoring the bacterial community structure established by the previous crop species within a growing season. Lastly, the C-S treatment had less bacterial diversity and richness compared to the Ca-S and S-S treatments, even at the pre-seeding treatment. Thus, soybean crops grown after corn may benefit from the addition of an inoculum that helps to promote bacterial diversity before the start of the growing season (Megali et al., 2015; Beirinckx et al., 2020).

### Fungal Community Diversity and Richness in the Bulk Soil and Soybean Rhizosphere Are Resilient to Crop Sequence

We showed that crop sequence did not significantly alter fungal community diversity and richness structure at all time-points. In a study that investigated fungal community composition in soils with varied cover crop treatments using a 3-year vegetable-cover crop rotation, it was reported that fungal community diversity was not affected by the identity, function, and diversity of cover crop species (Cloutier et al., 2020). In addition, it has been reported that rotation of different vegetables can have different effects on the diversity and richness of fungi in a continuous cropping matrix where rotation of celery improved the richness and diversity of fungi while beans and cabbage reduced the richness and diversity of fungi (Lyu et al., 2020). In addition, Lyu et al. (2020) reported that crop rotation can effectively reduce the number of culturable fungi in soil samples. Based on the above findings, fungal community diversity and richness may be influenced by crop-specific factors.

Studies that observed a significant effect of crop rotation on fungal community diversity and richness were long-term and spanned several growing seasons (Ding et al., 2018, Sommermann et al., 2018; Weihua and Qi-zhi, 2019). Indeed, a 12-year study that investigated changes in fungal community dynamics and diversity in the strawberry rhizosphere where soil samples were analyzed at years 0, 2, 4, 6, 8, 10, and 12, provided evidence that the diversity of soil fungi changes as the duration of continuous cropping increases (Weihua and Qi-zhi, 2019). An explanation for this could be the faster reproduction and turnover of bacteria compared to fungi in soil. Thus, extending the timeframe of the current study over multiple years may provide additional insights into the effect of long-term crop rotations on fungal community diversity and richness.

### Crop Sequence Did Not Alter Microbial Diversity in Both the Rhizosphere and Bulk Soil

In our study, the bacterial and fungal diversity of the bulk compared to the rhizosphere soil did not differ significantly based on the crop sequences tested. This finding is supported by a study done by Reddy et al. (2013), using corn and soybean, that showed minor changes in soil microbial communities in response to cropping sequence and significant differences between bulk and rhizosphere soils were not observed until the fourth year of the study. Hilton et al. (2013) reported that crop type did not have a significant effect on the overall bacterial community in the rhizosphere or the bulk soil in a shortened crop rotation of an oilseed rape study. In addition, given that rhizosphere soil members are horizontally transferred from the bulk soil it is likely that the environmental conditions and soil type of the field could be a driver of species diversity (Lloyd et al., 2016). Indeed, it has been shown that microbial community structure in the plant rhizosphere is primarily determined by soil composition and not by plant legacy since environmental conditions are one of the largest drivers in rhizosphere soil species diversity (Xue et al., 2018; Schmid et al., 2019). Therefore, we hypothesize that the duration of the crop sequence, as well as the relatively homogenous soil type found at the field site, provide some explanations for the similarities observed in the diversity of the fungal and bacterial communities between bulk and rhizosphere soils for all crop sequences. However, the current study did not investigate the soil type, moisture content, or nutrient profile. Future studies could combine soil analysis and metagenomics to provide additional evidence into the factors that drive species diversity within the bulk and rhizospheric soils.

### The Impact of Crop Sequence on Microbial Taxa in the Bulk and Rhizosphere Soil

Identification of core fungal species is essential for elucidating the ecology of microbial consortia and that microbes associated with a particular habitat are likely critical for the community function (Shade and Handelsman, 2012). As farmers are generally advised to practice crop rotation through changes in cropping sequence rather than continuously cropped soybean, we investigated the impact that continuous soybean sequence might have on microbial taxa. In this study, the most abundant bacterial phyla were consistent for all treatments regardless of the time-points and crop grown. The dominant bacteria phyla were the *Actinobacteria, Proteobacteria*, and *Acidobacteria.* These taxa are core bacterial taxa found in soybean and corn rotation fields (Compant et al., 2019; Chamberlain et al., 2020) and thus, it is expected that these were the dominant bacterial phyla in our study (Turley et al., 2020). At the phyla level, significant differences in the relative abundance of bacterial population for the C-S treatment were detected as the *Actinobacteria* and *Proteobacteria* were more abundant in the C-S crop sequence. This is in agreement with some studies that have reported that in the presence of commercial corn cultivars, there is an increase in the abundance of bacteria from the *Actinobacteria* and *Proteobacteria* phyla in the bulk soil due to the increase in the availability of labile organic carbon in the rhizosphere (Peiffer et al., 2013; Li et al., 2014; Aguirre-von-Wobeser et al., 2018). At the class level, the relative abundance of *Thermoleophila* (*Actinobacteria*) increased in the C-S sequence compared to the Ca-S and S-S crop sequences. This observation is in agreement with previous corn-soybean rotation studies that reported *Thermoleophilia* is generally more abundant in corn sequences (Hu et al., 2019). The current study showed that in an annual canola-soybean sequence, the bacterial community composition is similar to a continuous soybean sequence.

Averaging across all sampling time-points and crop sequences most reads belonged to OTUs assigned to the *Ascomycota, Mortierellomycota*, and *Basidiomycota.* This finding is in agreement with other studies that report members of the same three fungal phyla were the most abundant in canola, corn, and soybean crop rotation systems (Lay et al., 2018; Sommermann et al., 2018; Hu et al., 2019; Lyu et al., 2020). At the phylum level, there was no significant difference in relative fungal abundance based on crop sequence. In addition to our study, the *Mortierellomycetes*, *Sordariomycetes*, and *Tremellomycetes* have all been reported as the dominant fungal classes in other crop rotation studies (Maarastawi et al., 2018; Cloutier et al., 2020). For example, the *Mortierellomycetes* has been identified as a soybean-associated taxon and reported to increase in continuous soybean fields (Hu et al., 2018, 2019). In our study, the corn-soybean crop sequence had significantly fewer genera of the *Sordariomycetes* compared to the canola and continuously cropped soybean sequences. *Sordariomycetes* abundance was shown to be induced by legumes and showed reduced abundance in cornfield plots (Ai et al., 2018). The findings of the current study support a previous report that a corn-soybean sequence affected the fungal composition and altered the dominant genera in a short-term crop sequence study (Liu H. et al., 2019). Significant differences in fungal composition observed at the class but not phylum level is explained by a report that showed higher taxonomic levels give reliable assignment for ecological fungal taxa (Tedersoo et al., 2018). Taken together, in addition to longer crop rotations, investigating soil microenvironment will shed light on the biological functions of certain fungal species since changes in soil microenvironment caused by crop rotation has been shown to effectively improve physical and chemical properties of the soil, regulate soil fertility, and increase crop yield (Sommermann et al., 2018).

## Conclusion

Data from this study suggest that the preceding corn sequence affects both the relative abundance of bacterial and fungal communities within the soil. While our analysis focused on the impact of crop sequence over a single growing season, additional multi-year studies with other agriculturally important crops may provide the necessary insight into the long-term impacts of different crops on soil health at the microbe level. Our results provide a novel understanding of the microbial communities present in the bulk and rhizosphere soil and how these communities change over time due to crop sequence practices. Taken together, the findings of this study have broader agricultural implications on short-term and cropping sequence practices for those interested in soil and crop management.

## Data Availability Statement

The datasets presented in this study can be found in online repositories. The names of the repository/repositories and accession number(s) can be found below: https://www.ncbi.nlm.nih.gov/, PRJNA659713.

## Author Contributions

AB, JW, TK, WF, and MB conceived and designed the study. AB and JW performed the data analysis and analyzed the data. AB, JW, CM, YL, WF, and MB prepared and wrote the manuscript. YL designed and led the crop-sequence field experiment and contributed to the field sampling design. JW collected and analyzed soil samples. All authors read and approved the final manuscript.

## Conflict of Interest

The authors declare that the research was conducted in the absence of any commercial or financial relationships that could be construed as a potential conflict of interest.

## Footnotes

1 http://www.earthmicrobiome.org/

2 https://github.com/lh3/seqtk
