# Supplementary material for: Microbial Community Dynamics of Soybean (Glycine max) Is Affected by Cropping Sequence
Source: Front Microbiol. 2021 Feb 11;12:632280. doi: 10.3389/fmicb.2021.632280 (PMC7904696; doi:10.3389/fmicb.2021.632280)
Supplement: Supplementary file 1 [file Data_Sheet_1.docx]

**Supplementary Figures**


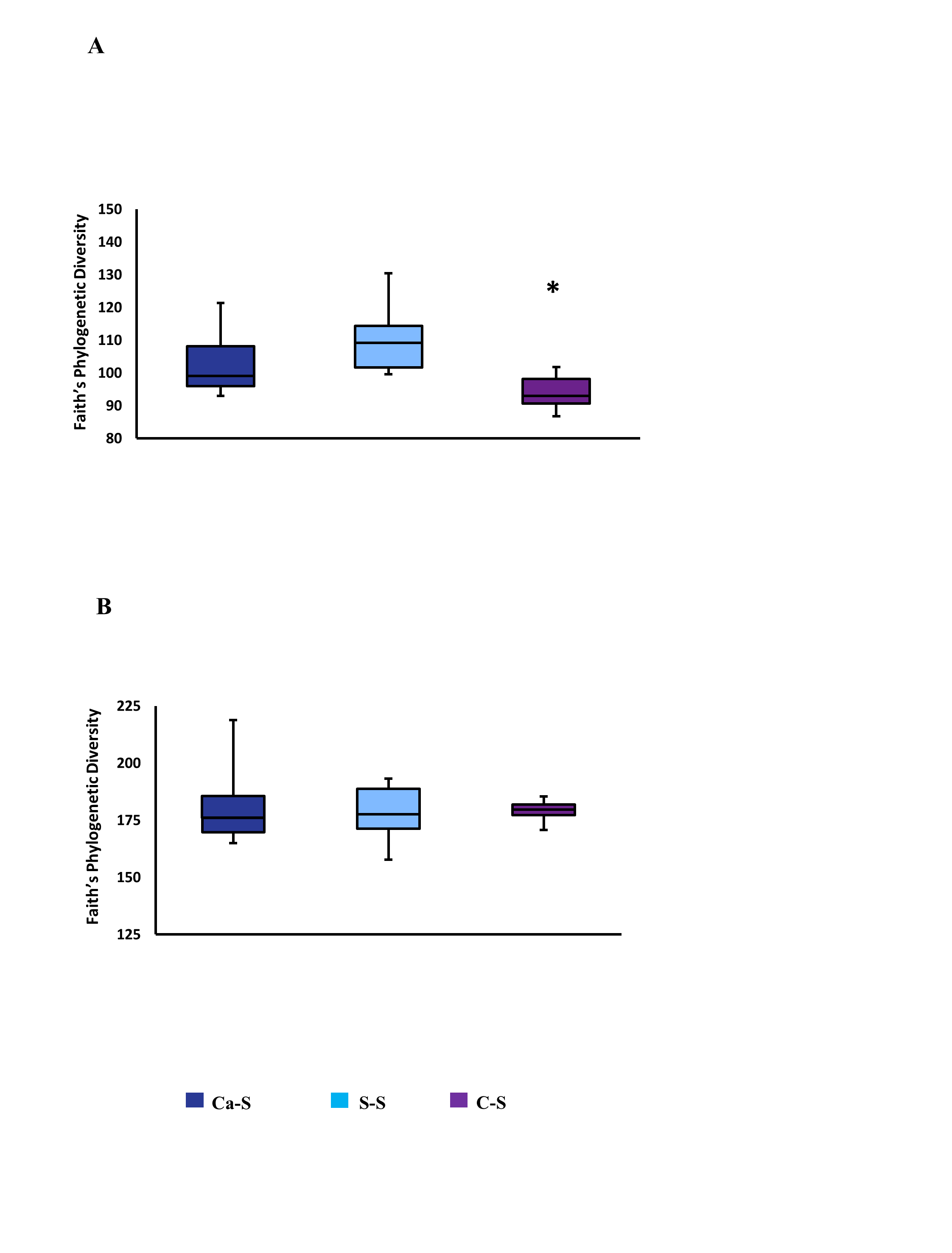


**Supplementary Figure 1**. Numerical quantification of microbial species richness represented by alpha diversity metric – Faith’s Phylogenetic Diversity – in bulk soil samples for all time points. The dark blue dots represent the canola-soybean sequence (Ca-S), light blue dots are the soybean-soybean sequence (S-S) and the purple dots are the corn-soybean sequence (C-S). Each boxplot represents an average of three biological replicates. A) Comparison of cumulative bulk soil samples of bacteria alpha diversity. As indicated with an asterisk, the C-S sample differed significantly less (P >0.05) from the Ca-S and S-S samples. B) A comparison of cumulative bulk soil samples of fungal alpha diversity showed that there was no statistical difference among treatments. Statistical tests were performed by the Kruskal-Wallis test.


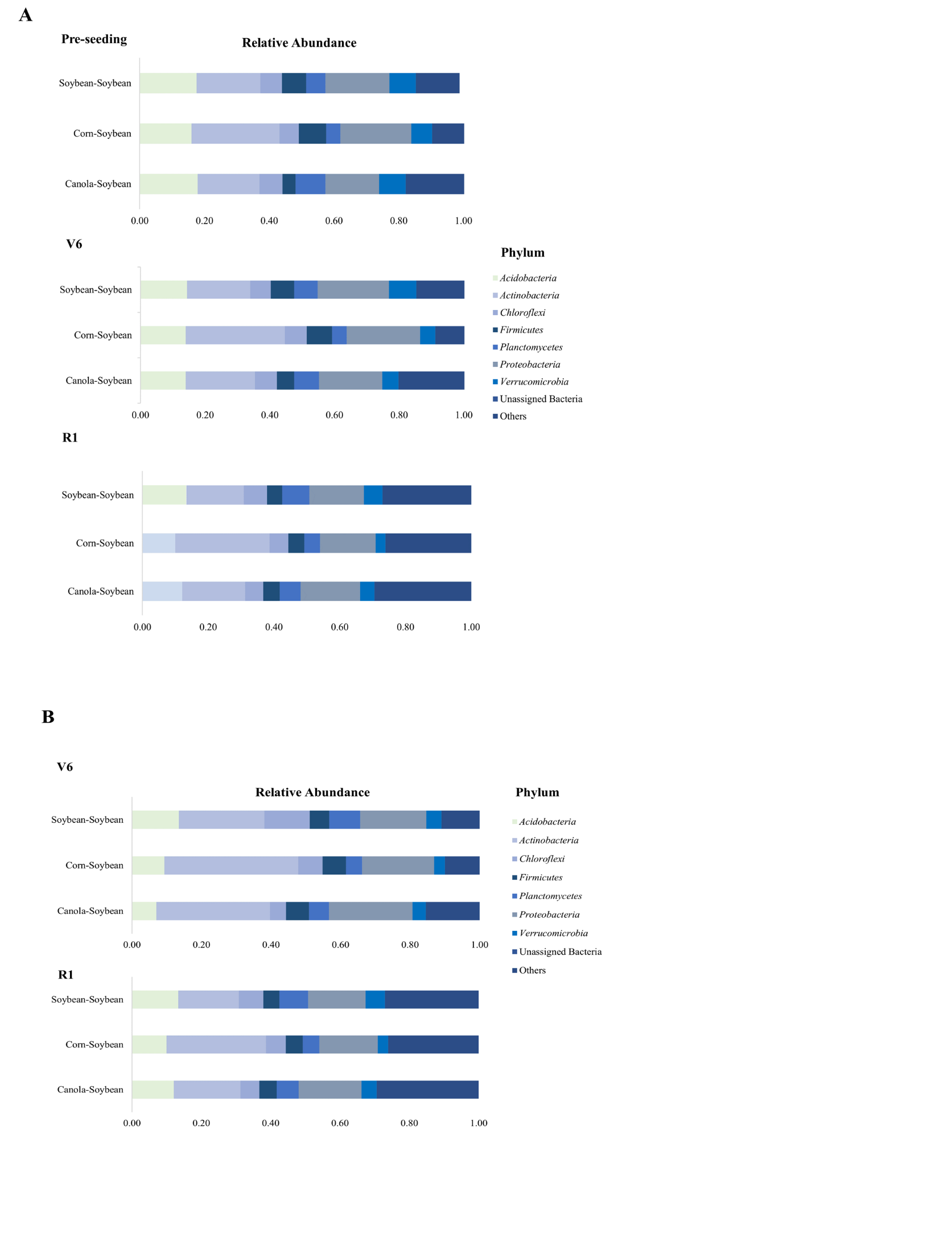


**Supplementary Figure 2.** Stacked bar plots of bacterial taxonomic composition showing shifts in major bacterial phyla in response to crop sequence treatments, during the soybean growing season in the bulk soil and rhizosphere soil. Each bar represents an average of three biological replicates. OTUs with similar species-level identity at 99% similarity in the GreenGenes database are merged here. Taxa with less than 1% mean relative abundance across the samples studied are combined and shown as others. A) Bar plots of bulk soil treatments of the relative abundance at the pre-seeding, V6, and R1 growth stages. B) Bar plots of rhizosphere soil treatments of the relative abundance at the V6 and R1 growth stages.


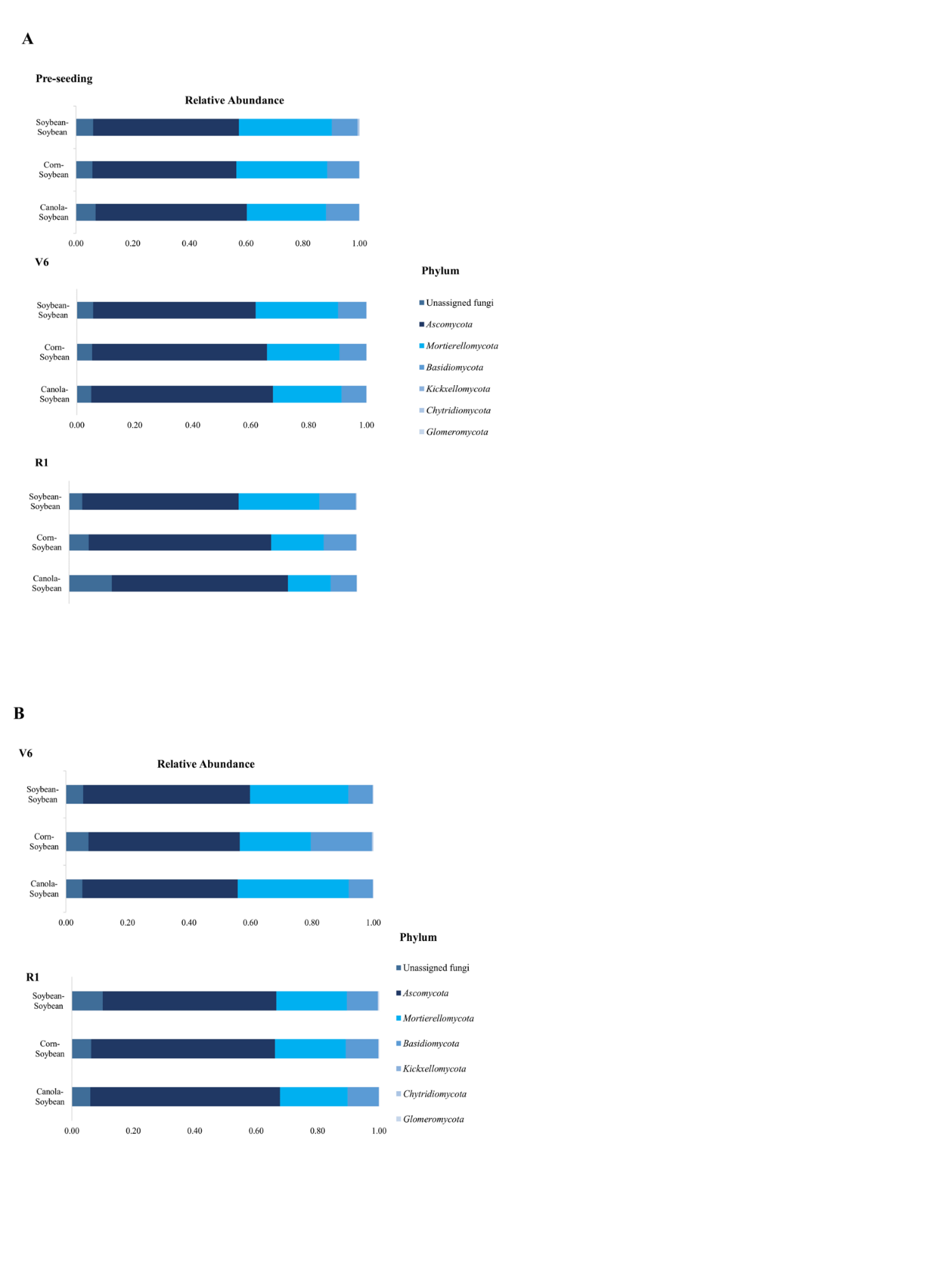


**Supplementary Figure 3**. Stacked bar plots of fungal taxonomic composition showing shifts in the major fungal phyla in response to crop sequence over the soybean growing season in the bulk soil and rhizosphere soil. Each bar represents an average of three biological replicates. OTUs with similar species-level identity at 97% similarity in the UNITE database are merged here. Taxa with less than 1% mean relative abundance across the samples studied are combined and shown as others. A) Bar plots of bulk soil treatments of the relative abundance at the pre-seeding, V6, and R1 growth stages. B) Bar plots of the soybean rhizosphere soil of the relative abundance at the V6 and R1 growth stages.


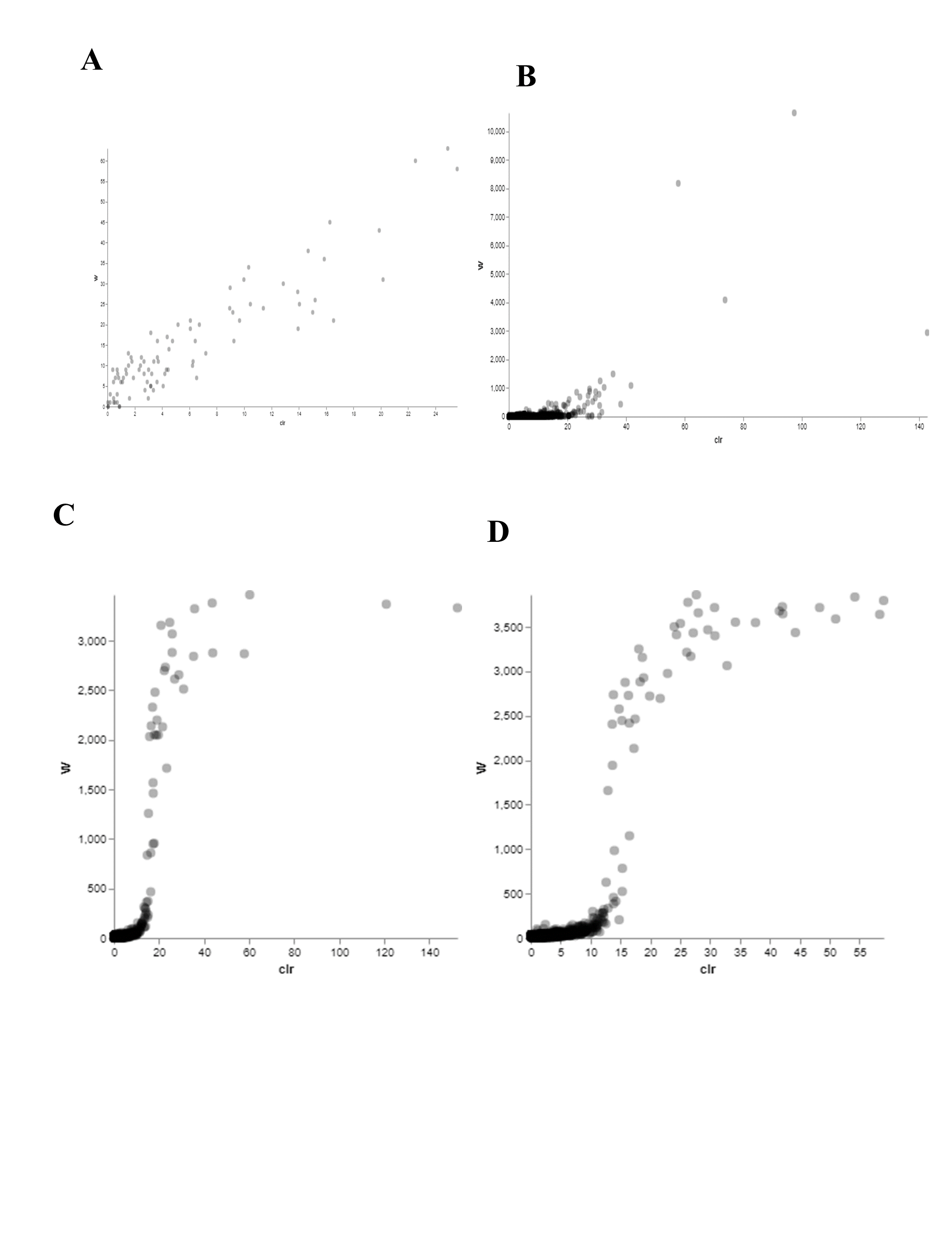


**Supplementary Figure 4**. Differentially abundant microbial taxa identified by ANCOM. F-statistics (clr) are represented on the x-axis and W-statistics on the y-axis. The F-statistics are a measure of the effect size differences between crop sequences, and the W-statistic is the strength of the ANCOM test. Volcano plot representation of ANCOM statistical analyses displaying the taxa that are significantly higher in all three crop sequences for A) Bacterial samples in the bulk soil. B) Bacterial samples in the rhizosphere soil. C) Fungal samples in the bulk soil D) Fungal samples in the rhizosphere soil.

**Supplementary Tables:**

**Supplementary Table 1:** *Permutational multivariate analysis of variance on soil bacterial community diversity for different crop sequences based on Bray-Curtis dissimilarity of samples in bulk and rhizosphere soil. Bulk soil and rhizosphere soil samples were combined for this analysis.*

| Group 1 | Group 2 | Sample Size | Permutations | pseudo-F | P-value |
| --- | --- | --- | --- | --- | --- |
| Canola-Soybean | Corn-Soybean | 30 | 999 | 3.027 | 0.001 |
| Canola-Soybean | Soybean- Soybean | 30 | 999 | 1.294 | 0.177 |
| Corn-Soybean | Soybean-Soybean | 30 | 999 | 6.006 | 0.001 |

**Supplementary Table 2:** *Permutational multivariate analysis of variance on soil fungal community diversity for different crop sequences based on Bray-Curtis dissimilarity of samples in bulk and rhizosphere soil. Bulk soil and rhizosphere soil samples were combined for this analysis.*

| Group 1 | Group 2 | Sample Size | Permutations | pseudo-F | P-value |
| --- | --- | --- | --- | --- | --- |
| Canola-Soybean | Corn-Soybean | 30 | 999 | 5.337 | 0.062 |
| Canola-Soybean | Soybean- Soybean | 30 | 999 | 2.346 | 0.128 |
| Corn-Soybean | Soybean-Soybean | 30 | 999 | 5.293 | 0.091 |

ANCOM test results for bacteria and fungi genera significantly enriched among crop sequences in the bulk soil and rhizosphere soil. Differentially abundant microbial taxa identified by ANCOM. W=W-statistics and the W-statistic is the strength of the ANCOM test for the tested number of species*.* K=kingdom, p=phylum, and c=class.

**Supplementary Table 3:** *ANCOM result to show differentially abundant bacterial taxa in the bulk soil*

| **W** | **Reject null hypothesis** | **Taxon** |
| --- | --- | --- |
| 63 | TRUE | k__Bacteria;p__*Armatimonadetes*;c__*Armatimonadia* |
| 60 | TRUE | k__Bacteria;p__*Actinobacteria*;c_*Actinobacteria* |
| 58 | TRUE | k__Bacteria;p__*Planctomycetes*; c__*Phycisphaerae* |

**Supplementary Table 4:** *ANCOM result to show differentially abundant bacterial taxon in the rhizosphere soil.*

| **W** | **Reject null hypothesis** | **Taxon** |
| --- | --- | --- |
| 10656 | TRUE | k__Bacteria;p__*Armatimonadetes*;c__*Armatimonadia* |

**Supplementary Table 5:** *ANCOM result to show differentially abundant fungal taxon in the bulk soil,*

| **W** | **Reject null hypothesis** | **Taxon** |
| --- | --- | --- |
| 3462 | TRUE | k__Fungi;p__*Ascomycota*;c__*Sordariomycetes* |
| 3379 | TRUE | k__Fungi;p__*Ascomycota*;c__*Sordariomycetes* |
| 3367 | TRUE | k__Fungi;p__*Ascomycota*;c__*Sordariomycetes* |
| 3330 | TRUE | k__Fungi;p__*Ascomycota*;c__*Eurotiomycetes* |
| 3321 | TRUE | k__Fungi;p__*Ascomycota*;c__*Eurotiomycetes* |
| 3183 | TRUE | k__Fungi |
| 3154 | TRUE | k__Fungi;p__*Ascomycota*;c__*Eurotiomycetes* |

**Supplementary Table 6:** *ANCOM result to show differentially abundant fungal taxon in the rhizosphere soil.*

| **W** | **Reject null hypothesis** | **Taxon** |
| --- | --- | --- |
| 3862 | TRUE | k__Fungi;p__*Ascomycota*;c__*Sordariomycetes* |
| 3837 | TRUE | k__Fungi;p__*Ascomycota*;c__*Sordariomycetes* |
| 3797 | TRUE | k__Fungi;p__Ascomycota;c__*Sordariomycete*s |
| 3777 | TRUE | k__Fungi |
| 3728 | TRUE | k__Fungi;p__*Ascomycota*;c__*Sordariomycetes* |
| 3718 | TRUE | k__Fungi;p__*Ascomycota*;c__*Eurotiomycetes* |
| 3718 | TRUE | k__Fungi;p__*Basidiomycota*;c__*Tremellomycetes* |
| 3679 | TRUE | k__Fungi;p__*Ascomycota*;c__*Leotiomycetes* |
| 3659 | TRUE | k__Fungi;p__*Ascomycota*;c__*Sordariomycete*s |
| 3648 | TRUE | k__Fungi |
| 3641 | TRUE | k__Fungi;p__*Ascomycota*;c__*Sordariomycete*s |
| 3590 | TRUE | k__Fungi;p__*Ascomycota*;c__*Leotiomycetes* |
| 3554 | TRUE | k__Fungi;p__*Ascomycota* |
| 3549 | TRUE | k__Fungi;p__*Ascomycota*;c__*Sordariomycetes* |
| 3539 | TRUE | k__Fungi |
| 3503 | TRUE | k__Fungi;p__*Basidiomycota*;c__*Agaricomycetes* |
